# Fishing Participation Explained Through an Extended Theory of Planned Behavior Model

**DOI:** 10.1007/s00267-026-02482-5

**Published:** 2026-04-30

**Authors:** North Joffe-Nelson, Adam Transue, Elizabeth Golebie, Joseph J. Parkos, Len M. Hunt, Carena J. van Riper

**Affiliations:** 1 Department of Natural Resources and Environmental Sciences, University of Illinois Urbana-Champaign, Urbana, IL USA; 2 Kaskaskia Biological Station, Illinois Natural History Survey, Prairie Research Institute, University of Illinois Urbana-Champaign, Sullivan, IL USA; 3 Centre for Northern Forest Ecosystem Research, Ontario Ministry of Natural Resources, Thunder Bay, ON Canada

**Keywords:** Environmental identity, Constraints, Recreation, Fishing, Outdoor recreation

## Abstract

Internal and external factors work in tandem to shape environmental behavior and guide strategies for natural resource management agencies, yet these factors are rarely integrated in empirical research. This study extended the Theory of Planned Behavior by incorporating constraints and environmental identity into a model to explain behavioral intentions among recreational anglers. Attitudes, perceived behavioral control, and environmental identity were strong correlates of environmental behavior, whereas constraints were weakly and negatively associated with intentions to go fishing the following year. Interestingly, environmental identity decreased intentions to go fishing while heightening positive attitudes towards fishing. These results provide a comprehensive perspective on a range of social psychological phenomena that work in conjunction to shape behavior and inform environmental education campaigns aimed at increasing participation in fishing.

## Introduction

Multiple theories guide environmental management research focused on understanding how humans cause, experience, and ultimately seek to address conservation problems. Knowledge of what drives behavioral engagement is crucial for promoting environmental sustainability; however, few studies have synthesized or empirically compared different theoretical approaches (Stern et al., 1999; Wall et al., 2007). Commonly utilized theories such as the Theory of Planned Behavior (TPB) (Fishbein & Ajzen, 2011; Yuriev et al., 2020) have demonstrated the value of attitudes, subjective norms and perceived behavioral control for the prediction and change of behavior but have also stirred ample discussion about the omission of other influential factors in TPB-guided research (Ajzen, 2020; Conner & Armitage, 1998). For example, an individual’s self-concept was absent from the original reasoned action approach, which has consequently motivated research to determine how identity-related constructs enhance the predictive capacity of behavioral models (Willis et al., 2020; Fielding et al., 2008). Meanwhile, other streams of research have shown that contextual factors, such as barriers, are well positioned to provide more comprehensive knowledge to inform environmental behavior change strategies (Champion & Skinner, 2008; Golebie et al., 2023; Shrestha & Burns, 2016). Systematic evaluations of the extent to which internal *and* external factors can extend the TPB are needed for theory building and testing in the social and behavioral sciences.

The application of the TPB to environmental behavior, such as participation in outdoor activities, remains underspecified in two important ways. First, the framework was developed and validated primarily in health and consumer behavior contexts, where identity and contextual barriers play comparatively minor roles. Extending it to environmental behavior, where both self-concept and structural access are known to shape engagement (Clayton & Czeller, 2023), therefore constitutes a meaningful theoretical expansion rather than a straightforward application. Second, prior extensions of the TPB have largely examined identity constructs and constraints in isolation rather than simultaneously (Moghimehfar et al. 2018; Rise et al. 2010), leaving open the question of whether these factors operate independently or interact in shaping behavioral intentions. Incorporating environmental identity and constraints alongside the core TPB variables in a single model would provide an empirical test of the relative and combined explanatory power of internal and external factors, therefore contributing to ongoing debates about relevant extensions to TPB used as a theory of environmental behavior (Sandberg & Conner, 2008).

Identifying the drivers of environmental behavior has direct relevance to the design of behaviorally informed management strategies for recreational fisheries and heeds a call to understand drivers of angler engagement as a vital component of stewardship of aquatic resources (Elmer et al. 2017; van Riper et al., 2019). Recreational anglers are a key constituency that has been studied through behavior change research, given their positive (Tufts et al., 2015; Landon et al., 2018) and negative (Golebie et al., 2022; Howell et al., 2015) impacts on waterways, as well as their financial contributions to resource management activities (Duda et al., 2022). Indeed, revenue from recreational fishing is a major source of support for natural resource management agencies, so decreases in fishing participation would constitute a major disruption to the management of aquatic ecosystems. Although declining trends in fishing participation across much of the United States and other post-industrial countries are well documented (Arlinghaus et al., 2015; Zhang et al., 2021), ramifications from these changes are still under study and need to be mitigated with deeper knowledge of the reasons underlying engagement in recreational angling (Birdsong et al., 2021). This changing pattern of environmental behavior, therefore, warrants research attention to inform resource management intervention strategies.

**Fig. 1:**
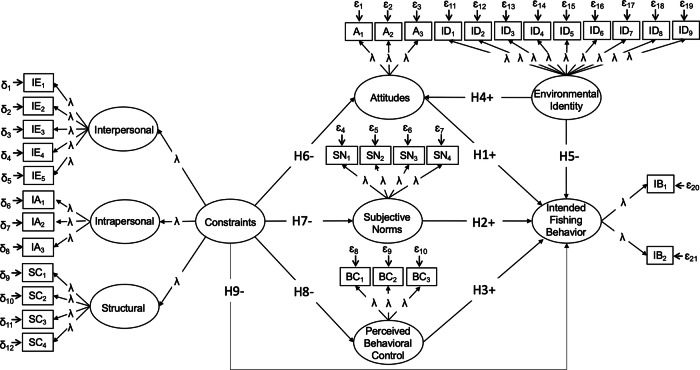
Hypothesized model showing predicted relationships among intended fishing behavior (i.e., fishing participation), attitudes, subjective norms, perceived behavioral control, environmental identity, and constraints. All latent variables are enclosed in circles and survey items are in boxes. All codes in the boxes correspond to questions detailed in Table 2. H1 through H9 are hypothesized paths. Note: ɛ represents expected measurement error for each endogenous question item; δ represents expected measurement error for exogenous variable items; λ represents factor loading scores for each question item

In this study, we evaluated the social psychological drivers of fishing participation and compared two models to determine how the addition of internal factors, such as environmental identity, and external factors, such as structural constraints, affected our ability to explain variance in the environmental behavior of fishing participation. The most extensive model incorporating all study variables was expected to account for the greatest variance in environmental behavior, thereby supporting decisions made by those tasked with managing fisheries resources amid changing trends in license sales. With theoretical guidance from the TPB, we hypothesized that constraints would negatively correlate with attitudes, subjective norms, perceived behavioral control, and intended behavior (H1–H4). We also hypothesized that each baseline TPB variable would be positively associated with intended fishing behavior (H5–H7). Finally, we hypothesized that environmental identity would positively predict both attitudes and behavioral intentions (H8 – H9), see Fig. 1.

## Theory of Planned Behavior Applied to Environmental Behavior

The TPB (Fishbein & Ajzen, 2011) has been used to explain the psychological drivers of behavioral intentions in an expansive body of previous research spanning disciplines such as environmental studies, health sciences, business and management, and education (Bosnjak et al., 2020; Conner & Armitage, 1998). Under the assumption that beliefs guide behavioral decision-making, the TPB posits that intentions are a direct antecedent of behavior and are positively influenced by an individual’s orientation towards the behavior (i.e., attitudes) and perceived social pressure (i.e., subjective norms). The perceived capability to perform a behavior (i.e., perceived behavioral control) is also theorized to be a key antecedent of behavioral intentions and a moderator of the previously described relationships (Ajzen, 2020). Numerous empirical investigations have shown that the TPB’s theoretical constructs that determine behavioral intentions are based on beliefs about attitudes, subjective norms, and perceived behavioral control, all of which have been effectively measured using reliable and valid survey scales (Coon et al., 2020; Fishbein & Ajzen, 2011).

The conceptual framework offered by the TPB can be applied to understand environmental behavior defined simply as actions that involve interaction with nature. Environmental behavior extends beyond pro-environmental action to include steps taken without explicit intention to improve or preserve the health of the natural world, including habits, purely recreational activities such as hiking, and consumptive activities such as fishing (Gatersleben, 2018; Steg & Vlek, 2009). For example, previous research on environmental behaviors such as hunting found that the TPB predictors explained 51% of the variance in deer hunting behavior in one year and 21% variance in the following year (Shrestha & Burns, 2016). Another study focused on pro-environmental activism incorporated the core TPB antecedents into a hierarchical multiple regression model along with general environmental attitudes. The authors found that the TPB explained an additional 32% of the variance in intentions to engage in environmental activism among students, in addition to the 19% explained by general environmental attitudes, thereby bolstering the model’s predictive capacity (Fielding et al., 2008). With adherence to the guidelines established by the TPB, such as compatibility between the behavior of interest and its antecedents (Ajzen, 2020), past work has argued this theory can explain high degrees of variance in predicting intentions to perform specific environmental behaviors over time (Armitage & Conner, 2001; Hagger & Hamilton, 2024), though not general classes of pro-environmental activity (Kaiser et al., 2005).

## Constraints on Environmental Behavior

Extensive research has been conducted on constraints, referred to as “barriers” (Kollmus & Agyeman, 2002; Champion & Skinner, 2008) and “contextual factors” (Steg & Vlek, 2009), which prevent a person from taking action (Moghimehfar et al., 2018). Our conceptualization draws from the leisure sciences literature that has examined how a range of factors limit participation in outdoor recreational activities (Sutton & Tobin, 2011; Wilhelm Stanis et al., 2009) and, therefore, the formation of preferences and accrual of nature-based benefits (Jackson, 1993). Previous research has considered constraints to comprise external factors, including structural and interpersonal conditions, alongside intrapersonal considerations processed by individuals (Crawford et al., 1991). An example of a structural constraint is a lack of infrastructure (e.g., a dock or an accessible shoreline) that enables fishing behavior. Interpersonal constraints refer to an individual’s social situation (e.g., a fishing club) and how people close to them may enable or impede behavior (e.g., not knowing people who fish), while intrapersonal constraints reflect an individual’s inner world (e.g., self-assessment of knowledge of angling) (Metcalf et al., 2015; Steg & Vlek, 2009). How individuals experience these constraints depends on both individual and systemic barriers (Stodolska et al., 2020), including factors such as personal identities and cultural backgrounds (Basto et al., 2025).

Researchers have posited that constraints interface with and extend TPB’s core constructs (Alexandris et al., 2022; Shrestha et al., 2012; Walker et al., 2007), as well as explain environmental behavior (Yoon et al., 2013). The concept of perceived behavioral control (PBC) has been likened to, but distinguished from barriers, because both relate to perceptions of access to an activity and the difficulty of a behavior (Moghimehfar et al., 2018). An individual’s PBC in part reflects perceived capabilities but also the adoption of strategies to overcome constraints. Using this logic, low PBC can constrain behavior because an individual may feel they cannot overcome existing barriers (Steg & Vlek, 2009); however, PBC is hypothesized to positively correlate with behavioral intentions related to the environment, whereas constraints are hypothesized to negatively correlate with these intentions (Yoon et al., 2013). The other core TPB concepts – attitudes and subjective norms – relate to internal and societal factors, respectively (Shrestha et al., 2012). These interrelationships have led authors to hypothesize negative associations between constraints and environmental behavior; however, the results have been mixed, suggesting that constraints either lead to or flow from TPB’s attitudinal variables. For example, an evaluation of two competing models that positioned constraints as either direct predictors of intentions or antecedents to the predictors of intentions of front-country campers from four protected areas within the Alberta, CA, park system found that constraints were an indirect predictor of behavioral intentions and a negative predictor of the behavioral antecedents specified in the TPB (Moghimehfar et al., 2018). We therefore expected constraints to negatively correlate with both intended fishing behavior and its attitudinal predictors according to the TPB.

## Environmental Identity

Our conceptualization of environmental identity is based on Clayton’s (2003) work, which posits that individuals form emotional and cognitive linkages with the environment that define who they are. The development of an environmental identity progresses over time through an individual’s upbringing, particularly during impactful early life experiences (Shin & van Riper, 2025). An individual’s self-perception of being connected to and dependent on nature leads to greater sensitivity and attention to environmental issues. In contrast to a socially oriented perspective whereby an individual develops a self-view in response to broader networks and groups (Stets & Burke, 2000), environmental identity is defined as “a belief that the environment is important to us and part of who we are” (Clayton, 2003, pp 45-46). This concept is specific to an individual’s perceived ties to nature and differs from moral inclinations to perform pro-environmental behaviors or the frequency with which they are performed. Environmental identity, however, is hypothesized to positively correlate with pro-environmental behavior (Clayton, 2003; Clayton & Czellar, 2023).

A long-standing body of research has shown that an individual’s personal association with and affinity for nature are likely to influence behavior and are consequently strong candidates for inclusion in an elaborated TPB model (Carfora et al., 2017; Moghimehfar et al., 2018). The effect of identity on behavior is well established (Burke & Stets, 2022) because identity is rooted in a role played, which, over time, manifests as an understanding of the self (Stryker, 2008). In other words, an individual is thought to engage in role-appropriate behavior to validate and experience harmony between their self-view and resulting behavior (Fielding et al., 2008). Concepts that parallel environmental identity have explained substantial variance in behavioral intentions (Dono et al., 2010; Sierra-Barón et al., 2023; Sparks & Shepherd, 1992). For example, environmental self-identity (Sparks & Shepherd, 1992) improved the explanatory power of a TPB model among science teachers from Turkey (Ateş, 2020), and a meta-analysis found that the inclusion of self-identity positively correlated with attitudes and increased explanation of variance in behavioral intentions (Rise et al., 2010). These studies argued that environmental identity directly and positively affected attitudes, subjective norms, and perceived behavioral control.

Although previous research has incorporated environmental identity to enhance knowledge of the predictors of pro-environmental behavior (e.g., Carfora et al., 2017), comparatively few studies have focused on explaining environmental behavior. This is a meaningful oversight in past work because some environmental behaviors may deepen the actor’s connection to nature while having negative environmental impacts (Gatersleben, 2018). Therefore, environmental identity may influence environmental behavior differently than it influences pro-environmental behavior. For example, some individuals with strong environmental identities may view certain environmental behaviors, such as fishing or hunting, as inherently harmful and may resist participating in such activities (Clayton & Czellar, 2023). However, others may view these behaviors as opportunities to deepen their connections with the environment and care for it (Shephard et al., 2023), leading to a positive relationship between environmental identity and environmental behavior. Research is therefore needed to determine whether the increased salience of environmental identities increases or decreases the performance of the specific environmental behavior of recreational angling.

## Methods

### Data Collection and Study Context

We surveyed adult residents who had fished recreationally in the previous 5 years (2019–2023) in the US state of Illinois (*n* = 1000). Respondents were recruited and incentivized to participate in the study by a company called Qualtrics in the summer of 2023. Quotas were applied to ensure the sample was geographically representative between rural and urban parts of the state. Geographic representation was important given that Illinois varies widely in population density from the urbanized Chicago area, where recreational fishing has declined most rapidly, to the agricultural and more forested downstate regions (Burkett & Winkler, 2019; Joffe-Nelson et al., 2025). The assembled database was screened for bot use and inattentiveness, including a “Captcha” requirement, two attention-check questions, and assessment of suspicious answers to open-ended questions. An exact response rate as not given by Qualtrics but in correspondence with the company we learned that the response rate was likely to be around 10%.

### Measurement

A survey questionnaire was developed to measure each theoretical construct using multi-item scales informed by previous research, see Appendix. Attitudes, measured using a scale adapted from Prinbeck et al. (2011), reflected the extent to which the individual believed that performing a behavior would yield utility. Next, subjective norms and perceived behavioral control were drawn from Fielding et al. (2008) to evaluate an individual’s beliefs about others’ normative perspectives on a behavior and the extent to which they believed they could, if desired, participate in fishing. The survey items in these three scales were tailored to the topic of recreational fishing. The environmental identity scale used in this study was developed by Clayton and Opotow (2003) and refined by Clayton (2021). In contrast, three dimensions of constraints from Fedler and Ditton (2001) and TenHarmsel et al. (2021) were used to assess reasons respondents may not have participated in recreational fishing. Specifically, intrapersonal constraints referred to psychological impediments emanating from the self (e.g., lack of knowledge, interest, skill), interpersonal constraints described those that were borne from other people (e.g., not knowing other people to fish with the respondent), and structural constraints were considered limitations that are external to the individual (e.g., one’s health, finances, lack of access). A three-item scale measuring intended behavior were created using guidance from Ajzen (2021), though one of the items was dropped from the model due to theoretical concerns.

All measures utilized in the study were evaluated for internal consistency using Cronbach’s alpha and McDonald’s Omega, and for convergent validity using average variance explained. Our survey scales were further assessed for normality, skewness, and kurtosis (see Appendix A), including Shapiro-Wilk tests (Shapiro & Wilk, 1965), which indicated non-normality for all variables (*p* > 0.01). Consequently, a robust maximum likelihood (MLR) estimation procedure was utilized to correct standard errors (Satorra & Bentler, 2001). Missing data accounted for 0.1% of the total dataset and were handled using the Full Information Maximum Likelihood Method (FIML) within the R package “Lavaan.” Analyses were conducted using R version 2023.12.0 (R Core Team, 2023).

### Analysis

**Fig. 2:**
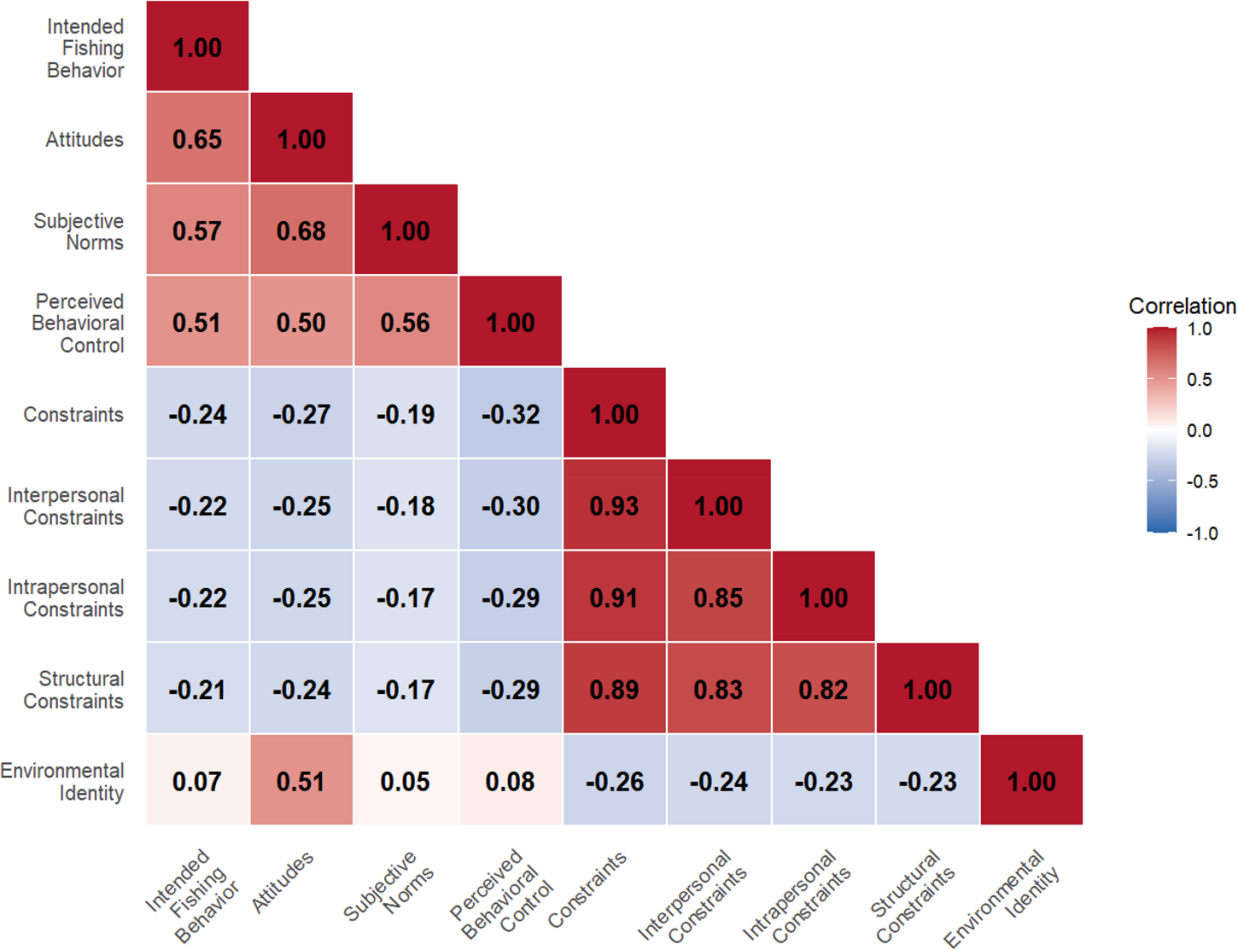
Zero-order correlations between pairs of variables included in the structural equation model of factors driving fishing participation among recreational anglers in IL

To test our hypotheses, we estimated two structural equation models. Model 1 included the core TPB variables of attitudes, subjective norms, and perceived behavioral control as predictors of intended fishing behavior (i.e., fishing participation), whereas Model 2 added both constraints and environmental identity as predictors. Each was tested using two-step structural equation modeling (Anderson & Gerbing, 1988), in which we first estimated a measurement model followed by a structural model. Model fit was evaluated using the chi-square (χ2) statistic and other fit statistics, given χ2’s sensitivity to sample size. Comparative fit index (CFI) values greater than 0.90, root mean square error of approximation (RMSEA) values less than 0.08, and standardized root mean square residual (SRMR) values less than 0.08 were considered acceptable (Hu & Bentler, 1999). All factor loading scores exceeded Hair et al.'s (2019) threshold of 0.40. The three dimensions of constraints were collapsed into a second-order variable due to multicollinearity (Schwarz et al., 2014) (see Fig. 2).

## Results

### Sample Composition

Our sample roughly matched the demographics of the state of Illinois, notwithstanding an overrepresentation of women compared to men, see Table 1. Survey respondents were 55.8% female, and most identified as White (83.3%), 13.7% as Black, and 5.1% as Hispanic or Latino (see Table 1). The average age was 43 years (*SD* = 15.40). As for the highest level of formal education, 38.6% reported having obtained a high school diploma or GED, 28.4% had completed at least a two-year college degree, 19.9% held a bachelor’s degree, and 13.1% held graduate degrees. A total of 27.9% reported an annual income of $49,999 or less, 31.8% made $50,000 to $99,999, and 32.7% reported earning between $100,000 and $199,999. Respondents reported averages of fishing for 16.7 years and 35 days per year (*SD* = 55.1) with a just above average skill level (*M* = 3.3, *SD* = 1.0). Over half (56.4%) fished from the shoreline or dock (including wading), while only 15.3% fished from a boat. Just over one quarter (28.3%) spent equal amounts of time adopting these different fishing modes. The most targeted species were catfish (genus *Ictaluridae*) (46.2%), carp (*Cyprinus carpio*) (37.5%), largemouth bass (*Micropterus nigricans*) (34.7%), bluegill (*Lepomis macrochirus*) (32%), and smallmouth bass (*Micropterus dolomieu*) (25.2%).

**Table 1:** Socio-demographic, previous experience, and descriptive characteristics of recreational anglers in Illinois, US (*n* = 1000)

Variable	*N* (%)
Age [M, SD]	43 (15.4)
Gender	
Male	438 (43.8)
Female	558 (55.8)
Non-binary/third gender	4 (0.4)
Education	
Some high school	33 (3.3)
High school graduate or GED	353 (35.3)
Two-year college degree	284 (28.4)
Bachelor’s degree	199 (19.9)
Graduate degree	131 (13.1)
Annual Household Income	
Less than $24,999	119 (11.9)
$25,000 to $49,999	160 (16.0)
$50,000 to $99,999	318 (31.8)
$100,000 to $149,999	198 (19.8)
$150,000 to $199,999	129 (12.9)
$200,000 and over	70 (7.0)
Prefer not to answer	6 (0.6)
Race^a^	
White	833 (83.3)
Black or African American	137 (13.7)
Asian	13 (1.3)
American Indian or Alaska Native	18 (1.8)
Native Hawaiian or other Pacific Islander	1 (0.1)
Hispanic or Latino	51 (5.1)
Other	2 (0.2)
Skill in relation to other anglers [*M, SD*]	3.3 (1.0)
Total days fishing in the previous 12 months^b^ [M, SD]	35.0 (55.1)
Total years of experience	16.7 (17.7)
Fishing Mode	
Shoreline or dock (including wading)	564 (56.4)
From a boat	153 (15.3)
About an equal amount of time fishing from boats and shorelines	283 (28.3)
Most commonly targeted species^a^	
Catfish	457 (46.2)
Carp	371 (37.5)
Largemouth bass	343 (34.7)
Bluegill	316 (32.0)
Smallmouth bass	249 (25.2)

^a^Respondents could check all that applied so column totals may not equal 100%

^b^Estimate refers to fishing days in 2023–2024

### Model Results

All survey scales evaluated in our study were first validated using confirmatory factor analysis (see Table 2). We also considered the relative performance between a traditional TPB model (Model 1) and an elaborated TPB model that included environmental identity and constraints. First, we found good fit for Model 1 (χ^2^ = 149.662, df=48, *p* = < 0.001; CFI = 0.975; RMSEA = 0.046; SRMR = 0.030) and partial support for our hypotheses. Specifically, results from our structural regression model illustrated that attitudes (*β* = 0.504, *p* < 0.001) (H1) and perceived behavioral control (*β* = 0.140, *p* = 0.003) (H3) were positively associated with intended fishing behavior whereas subjective norms had no significant correlation (*p* = 0.343).

**Table 2:** Confirmatory factor analysis results and descriptive statistics including mean values and standard deviations (SD) for survey items that measured intended fishing behavior, attitudes, subjective norms, perceived behavioral control, interpersonal constraints, intrapersonal constraints, structural constraints, and environmental identity among recreational anglers in Illinois, USA (*n* = 1000)

Scale items		λ	Mean (SD)
Intended Fishing Behavior (α = 0.858, Ω=0.858)			
IB_1_	Please rate your intention to make time for fishing in the next 12 months^a^	0.837	3.82 (1.08)
IB_2_	How strongly do you intend to make time for fishing over the next 12 months^a^	0.898	3.79 (1.06)
Attitudes^b^ (α = 0.688, Ω=0.695)			
A_1_	Fishing offers many benefits to me	0.723	3.96 (0.87)
A_2_	Fishing is generally a pleasant experience	0.605	4.28 (0.80)
A_3_	The practice of fishing teaches me useful skills	0.635	4.08 (0.83)
Subjective Norms^b^ (α = 0.737, Ω=0.740)			
SN_1_	If I engage in fishing, people who are important to me would approve	0.623	3.96 (0.96)
SN_2_	Most people who are important to me think that participation in fishing is desirable	0.728	3.94 (0.90)
SN_3_	Most people that I know are encouraging of my participation in fishing	0.745	3.90 (0.92)
Perceived Behavioral Control^b^ (α = 0.746, Ω=0.750)			
BC_1_	Whether or not I can go fishing is largely within my own control	0.506	4.12 (0.88)
BC_2_	For me, going fishing is easy	0.625	3.96 (0.99)
BC_3_	If I wanted to, I could easily find time to go fishing	0.670	3.99 (0.97)
BC_4_	I believe I have the ability to go fishing as much as I want	0.724	3.87 (1.03)
Interpersonal Constraints^b^ (α = 0.802, Ω=0.801)			
IE_1_	The people I know don’t have time to fish	0.616	2.98 (1.17)
IE_2_	The people I know don’t have money to fish	0.686	2.59 (1.210)
IE_3_	The people I know are not interested in fishing	0.666	2.80 (1.183)
IE_4_	The people I know don’t feel it’s appropriate to fish	0.669	2.41 (1.153)
IE_5_	I don’t know other people I can fish with	0.700	2.52 (1.231)
Intrapersonal Constraints^b^ (α = 0.734, Ω=0.746)			
IA_1_	I have a lack of information about fishing opportunities in Illinois	0.741	2.51 (1.169)
IA_2_	I have difficulty understanding fishing regulations	0.756	2.39 (1.162)
IA_3_	I have a lack of interest in fishing	0.607	2.00 (1.159)
Structural Constraints^b^ (α = 0.679, Ω=0.682)			
SC_1_	Fishing facilities are too crowded	0.523	2.90 (1.085)
SC_2_	I have too many family and/or work commitments	0.519	3.09 (1.267)
SC_3_	There are not enough fish for me to catch	0.613	2.48 (1.187)
SC_4_	I don’t have access to fishing opportunities	0.699	2.59 (1.206)
Environmental Identity^b^ (α = 0.880, Ω=0.880)			
ID_1_	I like to spend time outdoors in natural settings (such as woods, mountains, rivers, parks, lakes or beaches, gardens)	0.739	4.24 (0.792)
ID_2_	I think of myself as a part of nature, not separate from it	0.623	4.03 (0.950)
ID_3_	I feel comfortable out in nature	0.686	4.25 (0.851)
ID_4_	When I am upset or stressed, I can feel better by spending some time outdoors surrounded by nature	0.666	4.19 (0.902)
ID_5_	I enjoy encountering elements of nature, like trees and grass, even when I am in a city setting	0.679	4.21 (0.877)
ID_6_	Learning about the natural world should be part of everyone’s upbringing	0.648	4.19 (0.895)
ID_7_	If I could choose, I would prefer to live where I can have a view of the natural environment, such as trees or fields	0.661	4.16 (0.922)
ID_8_	An important part of my life would be missing if I was not able to get outside and enjoy nature from time to time	0.658	4.17 (0.919)
ID_9_	I feel refreshed when I spend time in nature	0.691	4.32 (0.789)

λ=factor loading score, α***=***Cronbach’s alpha, and Ω=MacDonald’s omega

^a^Survey items were presented on a Likert scale where 1 = “Not at all intend” and 5 = “Very strongly intend”

^b^Survey items were presented on a Likert scale where 1 = “Strongly Disagree” and 5 = “Strongly Agree.”

**Fig. 3:**
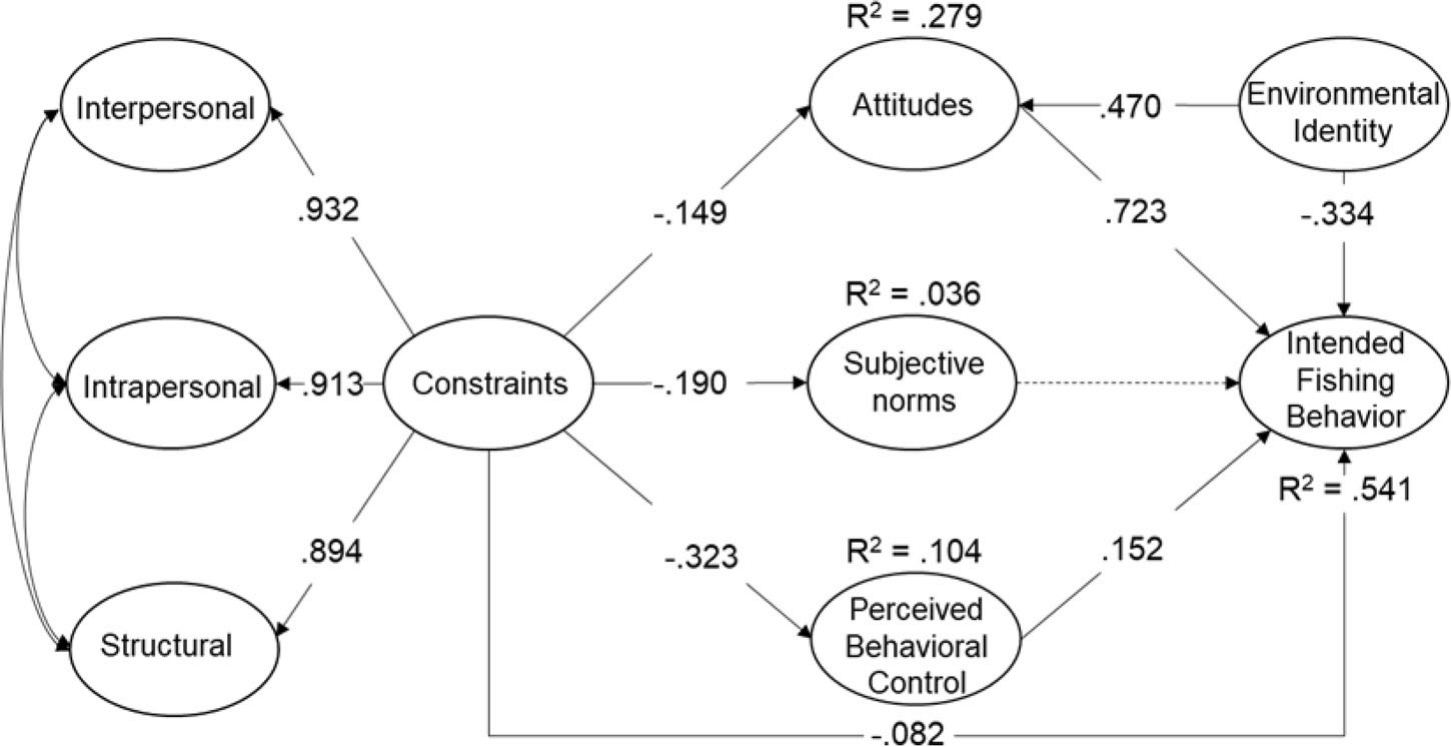
Results from a latent variable path model of relationships among attitudes, subjective norms, perceived behavioral control, environmental identity, and a second order model of constraints that included three dimensions. Beta (β) values are placed on the paths between significantly correlated variables and non-significant paths are shown using dotted lines

After incorporating internal and external predictors, including environmental identity and constraints in Model 2, we observed acceptable model fit for the measurement model (χ2 = 1316.595, df=467, *p* = <.001; CFI = .931; RMSEA = .043; SRMR = .046) and structural models, with the exception of SRMR (χ^2^ = 1548.101, df=479, *p* < .001; CFI = .913; RMSEA = .047; SRMR = 0.082) (see Fig. 3). As with Model 1, attitudes (*β* = .470, *p* < .001) (H1) and perceived behavioral control (*β* = .152, *p* = .014) (H3) directly affected intended fishing behavior, whereas subjective norms did not (*p* = .968) (H2). Environmental identity also exerted positive effects on attitudes (*β* = .470, *p* < .001) (H4) but negative effects on behavior (*β* = -.334, *p* < .001) (H5), as hypothesized. Constraints had a minimal direct effect on intended fishing behavior (*β* = -.082, *p* = .030) (H9). As expected, constraints were negatively associated with attitudes (*β* = -.149, *p* < .001) (H6), subjective norms (*β* = -.190, *p* < .001) (H7), and perceived behavioral control (*β* = -.323, *p* < .001) (H8). Model 2 accounted for a moderately high degree of variance in the environmental behavior of intentions to fish in the next year (R^2^ = .541), moderate variance in attitudes (R^2^ = .279), and low variance in perceived behavioral control (R^2^ = .104) and subjective norms (R^2^ = .036).

The next step was to compare the baseline TPB model (Model 1) against the model including constraints and environmental identity (Model 2) to evaluate the effects of incorporating internal and external factors into a well-established social psychological theory (see Table 3). Results suggested that TPB was an effective theory, accounting for a moderate degree of variance in environmental behavior. The additions of environmental identity and constraints offered marginal increases in explanatory power (change (Δ) in R^2^ = .07). However, including these constructs provided insight into behavioral antecedents, particularly attitude formation, which was the strongest driver of intended fishing behavior.

**Table 3:** Comparison between a classical and elaborated Theory of Planned Behavior (TPB) models including the R^2^ values for all dependent variables and the change (Δ) in R^2^ values

Dependent variables	R^2^ values in a classical TPB model (Model 1)	R^2^ values in an elaborated TPB model (Model 2)	Δ R^2^
R^2^ Intended fishing behavior	0.475	0.541	0.066
R^2^ Attitudes	-	0.279	-
R^2^ Subjective norms	-	0.036	-
R^2^ Perceived behavioral control	-	0.104	-

The indirect effects of constraints on intended fishing behavior were also calculated using a mediation analysis (see Table 4). This analysis indicated significant indirect effects of constraints on intended behavior through attitudes (*β* = −0.108 *p* = .009) as well as perceived behavioral control (*β* = −0.049 *p* = .022). The total effect of constraints on intended behavior (*β* = −0.238, *p* < .001) was larger than the indirect effect (*β* = −0.156, *p* < .001), indicating partial mediation.

**Table 4:** Results of a mediation analysis showing indirect effects of constraints on intended fishing behavior

Pathway	β	SE	*z*	*p*	95% CI
Constraints → attitudes → behavior	−0.108	0.075	−2.612	0.009	[−0.345, −0.049]
Constraints → norms → behavior	0.001	0.040	0.041	0.968	[−0.077, 0.080]
Constraints → PBC → behavior	−0.049	0.039	−2.293	0.022	[−0.165, −0.013]
Total indirect effect	−0.156	0.074	−3.852	<0.001	[−0.430, −0.140]
Total effect	−0.238	0.086	−5.046	<0.001	[−0.603, −0.266]

*CI* confidence interval

## Discussion

This study assessed the social psychological drivers of behavioral intention among recreational anglers in Illinois, USA. A moderate degree of variance (54.1%) was accounted for in intended fishing behavior using the TPB and two theoretical extensions. Attitudes towards fishing and environmental identity were the most strongly associated with intentions. Therefore, we suggest that agencies focus on targeting these behavioral correlates to increase fishing participation. An angler’s belief about their ability to fish (i.e., perceived behavioral control) was a significant but less influential psychological correlate of intended behavior, which aligns with previous research (Crandall et al., 2018). That is, the more anglers believe they are capable, the more likely they were to report higher intentions to fish, but only to marginal degrees. High values for perceived behavioral control indicated that anglers in our sample had already taken steps to enable their participation in fishing.

The tripartite structure of attitudinal variables in the TPB was helpful in explaining behavior, but environmental identity also contributed to explaining both behavioral intentions and attitudes. Although previous research has pointed to self-identity, past behavior, and concepts of emotion as potential enhancements to the TPB (Ajzen, 2020; Coon et al., 2020; Fielding et al., 2008; Rise et al., 2010), fewer studies have empirically tested these propositions (for an exception, see Moghimehfar et al. (2018)). The study of identity has predominantly driven improvements in predictive power (Rise et al., 2010), but less is known about environmental identity and its influence on the formation of environmental behavior (Dono et al., 2010). We therefore focused on understanding identity rooted in individuals’ interactions with nature (Stets & Biga, 2003; Van der Werff et al., 2014). Interestingly, environmental identity was negatively correlated with intentions to fish and positively correlated with attitudes towards fishing. It could be that individuals with strong environmental identities seek numerous ways to spend time outdoors and therefore dedicate a smaller share of that time to go fishing (Ditton & Sutton, 2004). Alternatively, people with strong environmental identities may be uncomfortable with potential animal welfare impacts on fishing (Arlinghaus et al., 2012) and therefore are more likely to lapse in their own participation, while still holding positive attitudes towards fishing in general (Gamborg & Jensen, 2017).

We observed that constraints were both directly and indirectly associated with lower intended fishing behavior, suggesting that greater perceived barriers were associated with belief systems less conducive to behavioral engagement (Moghimehfar et al., 2018; Shrestha & Burns, 2016). There were three significant barriers faced by anglers that agencies can help negotiate and overcome, including interpersonal factors (e.g., not knowing other anglers), intrapersonal factors (e.g., lacking information or knowledge), and structural factors (e.g., limited time and opportunities to fish). Evidence-based fisheries management decisions are needed to respond to these results and adopt a multifaceted approach to address each category of barriers faced by anglers (Cooke et al., 2024). These findings complement calls in the human dimensions literature for fisheries managers to move beyond biological assessments toward socially informed approaches that account for the psychological and contextual factors shaping angler behavior (Schroeder et al., 2018; Elmer et al. 2017).

Our results suggest that attitudes and environmental identity are particularly promising leverage points for agencies designing outreach and engagement strategies, as these constructs explained substantially more variance in behavioral intentions than structural constraints alone.

The TPB variables mediated the relationship between constraints and intended behavior, which is consistent with past research (Moghimehfar et al., 2018; Steg & Vlek, 2009). While other studies have noted that adding constraints accounts for an additional 10% of variance in behavioral intentions (Moghimehfar et al., 2018), the minimal direct association of constraints on intended behavior in our model yielded a much more modest increase in explanatory power. This may reflect our chosen population of current and former anglers, a group that has already demonstrated the capacity to navigate constraints on their fishing behavior. The role of constraints would likely have differed had our sample prioritized anglers who had lapsed in participation for more extended periods or individuals who had not previously gone fishing, thereby representing new user groups. Additionally, we used constraints as a basis for measuring contextual barriers. In contrast, the leisure constraints literature has aimed to account for a broader, more deeply seated range of behavioral inhibitors, such as socialization during childhood or a distaste for the activity (Crawford et al., 1991). Additionally, we did not include constraint negotiation as a concept in our modeling, which accounts for how well individuals cope with barriers to a given behavior (Jun & Kyle, 2011). Our study nevertheless suggests that contextual factors do not constitute a substantial barrier to intended participation in fishing, unlike identity.

We engaged anglers who had fished at least once in the past five years and therefore did not include individuals who had never fished. The minimal role of constraints in our modeling might reflect the composition of our sample, in that previous anglers have already negotiated constraints to participate in the activity. Similarly, our respondents likely had more positive views towards fishing than the public at large. Had we surveyed both anglers and non-anglers, a broader range of concerns, such as animal welfare (Arlinghaus et al., 2007), might have yielded different relationships between environmental identity and attitudes, while shedding light on changing ideals related to environmentalism. Future research should explore the identities, attitudes, and constraints to fishing faced by non-anglers to elucidate an understanding of how new anglers are recruited (Smith et al., 2023).

Although quotas were applied to ensure geographic representation between urban and rural parts of Illinois, we did not estimate separate models for urban and rural anglers, nor did we disaggregate findings by gender or race. Given the complexity of the elaborated TPB model tested here, introducing additional subgroup analyses risked compromising model stability and exceeded the scope of the current study. However, urban and rural anglers likely differ meaningfully in their environmental identities, access to fishing opportunities, and the variety of constraints they face. Meanwhile, prior research has documented substantial gender and racial differences in fishing-related constraints and participation patterns (Burkett & Carter, 2022; Toth & Brown, 1997). Future research should therefore examine whether the relationships identified here hold across these subgroups, as such analyses would yield more targeted insights for agencies seeking to address participation disparities among underrepresented anglers. Target groups such as younger audiences, women, more racially and ethnically diverse people, or immigrants could be engaged in discussions about their specific constraints to assist agencies in strategy development (Basto et al., 2025). Lastly, the elaborated TPB model, including constraints and identity, had an SRMR of 0.082, which exceeded the commonly used cutoff of 0.08 by only a marginal degree, so we proceeded to interpret the results.

There are important considerations related to our methodology. Our sample was generated from an online pool of people who were paid to participate in research, rather than relying on a random sample of anglers who had purchased fishing licenses. We adopted this approach in response to previous research that has called for adaptations in response to declining response rates (Stedman et al., 2019; Wardropper et al., 2021). Because we did not use a random sample, we cannot make inferences about the overall population of Illinois anglers. However, this limitation was a worthwhile tradeoff because our focus was on understanding relationships among internal and external drivers of behavior. One opportunity presented by our research approach was recruiting a greater proportion of recreational anglers who were underrepresented in databases of license-holding anglers. For instance, 19.6% of anglers in this study did not purchase a fishing license. Also, the sample used for this study included more females and more racially diverse individuals than previous fisheries research that has drawn from license sale databases (Golebie et al., 2021) and relied on angler surveys at boat ramps (van Riper et al., 2019). Although respondents were those who had previously gone fishing, our results may offer insights into the experiences of these groups that can be leveraged to further increase their participation and recruit new anglers.

## Environmental Management Implications

A common management goal for recreational fisheries in the U.S. Midwest is to promote fishing participation ensuring public access to natural resources. Fishing offers numerous benefits including supporting public health (Wang et al., 2025), fostering positive human-nature relationships (Shephard et al., 2023), and generating funding for conservation (Kopaska, 2026). Increased fishing participation can also bring challenges such as harvest pressure (Embke et al., 2019), the spread of aquatic invasive species (Weir et al., 2022), and habitat disturbance (Lewin et al., 2006). However, management agencies are well equipped to respond to these challenges while also seeking to boost low participation rates. Further, the benefits of fishing can be extended by cultivating anglers who are informed, conservation-minded stewards of aquatic resources (van Riper et al., 2021).

Understanding attitudinal and identity-based drivers of angler behavior is increasingly recognized as foundational to designing interventions that build conservation-minded participation rather than simply increasing engagement (Schroeder et al., 2018; van Riper et al., 2019; Hunt et al., 2013). Our results suggested that higher levels of environmental identity were associated with lower levels of fishing participation. Individuals with strong environmental identities may have ethical concerns about fish health (Arlinghaus et al., 2007), however this is unlikely to be the case in our sample given the strong positive correlation between identity and attitudes towards fishing. Rather, individuals with environmental identities may have lower intentions to fish because they have higher intentions to also engage in a variety of other outdoor activities. Retaining these individuals as recreational anglers is important because they help foster stewardship norms among the angling community. They may also serve as some of the easiest candidates for agencies to recruit or retain in service of increasing funding conservation. To appeal to individuals with strong environmental identities, campaigns designed to increase fishing participation should emphasize the unique opportunity fishing presents to connect with nature (Shephard et al., 2024) and the conservation outcomes that are made possible from license purchases (Kopaska, 2026).

Further, our results offer insights as to how agencies can help anglers negotiate constraints to fishing. We found that constraints were negatively associated with attitudes, norms, and perceived behavioral control, which in turn were strongly associated with fishing intentions. Therefore, constraints need to be addressed not only to enable fishing itself, but even to enable positive attitudes and norms towards fishing. We found that all three types of constraints were relevant: intrapersonal, interpersonal, and structural. Intrapersonal constraints can be addressed by providing resources to help anglers build fishing skills and navigate regulations. For example, fishing workshops can help prospective anglers build confidence and negotiate intrapersonal constraints related to low knowledge while also instilling conservation behaviors (Delle Palme et al., 2016; Morales et al., 2020). These programs can also help anglers navigate interpersonal constraints by helping anglers build a fishing community. Finally, structural constraints are often rooted in a lack of access to fishing locations that meet angler needs. Improving access remains a priority in many agency strategic plans and can be expected to lead to not only higher rates of fishing participation, but more positive attitudes and norms towards fishing.

## Conclusion

This study was developed to understand how an array of psychological factors shapes intended fishing behavior, including attitudinal variables from the Theory of Planned Behavior, environmental identity, and constraints. Attitudes, perceived behavioral control, and environmental identity were the strongest internal correlates of future fishing behavior. At the same time, constraints were less influential in explaining behavioral intentions but valuable for understanding attitude formation. Environmental identity was negatively associated with intentions to fish while being positively associated with respondents’ positive attitudes towards fishing as a practice. Environmental management in the United States is highly contingent upon the popularity of recreational fishing and the behavior of aquatic resource users and would therefore benefit from the insights generated in this study. Consequently, we show that psychologically oriented factors, including attitudes, environmental identity, and perceived behavioral control, directly correlate with angler behavior, whereas constraints are more associated with attitudes regarding fishing as a practice.

## Supplementary information

Appendix A

R3_questionnaire

## Data Availability

Data used in this study is not made available because work is ongoing that utilizes this dataset.
