# Supplementary material for: Fishing Participation Explained Through an Extended Theory of Planned Behavior Model
Source: Environ Manage. 2026 Apr 30;76(6):179. doi: 10.1007/s00267-026-02482-5 (PMC13133205; doi:10.1007/s00267-026-02482-5)
Supplement: Supplementary file 1 — Appendix A [file 267_2026_2482_MOESM1_ESM.docx]

**Appendix A**

**Table 1.**

*Results showing normality, skewness, and kurtosis for social psychological drivers of intended fishing behavior among recreational anglers in Illinois, USA (n = 1,000)*

| **Variable** | **Shapiro–Wilk** | **Skewness** | **Kurtosis** |  |
| --- | --- | --- | --- | --- |
| **Intended Behavior** | |  |  |  |
| Please rate your intentions to make time for fishing in the next 12 months | | 0.86* | -0.69 | -0.43 |
| How strongly do you intend to make time for fishing over the next 12 months | | 0.87* | -0.68 | -0.20 |
| **Attitudes** |  |  |  |  |
| Fishing offers many benefits to me | 0.84* | -0.85 | 1.04 |  |
| Fishing is generally a pleasant experience | 0.78* | -1.07 | 1.13 |  |
| The practice of fishing teaches me useful skills | 0.83* | -0.74 | 0.44 |  |
| **Subjective Norms** |  |  |  |  |
| If I engage in fishing, people who are important to me would approve | 0.85* | -0.69 | -0.09 |  |
| Most people who are important to me think that participation in fishing is desirable | 0.86* | -0.55 | -0.09 |  |
| Most people that I know are encouraging of my participation in fishing | 0.86* | -0.60 | 0.11 |  |
| **Perceived Behavioral Control** |  |  |  |  |
| Whether or not I can go fishing is largely within my own control | 0.81* | -1.01 | 0.91 |  |
| For me, going fishing is easy | 0.85* | -0.72 | -0.19 |  |
| If I wanted to, I could easily find time to go fishing | 0.84* | -0.80 | 0.07 |  |
| I believe I have the ability to go fishing as much as I want | 0.86* | -0.73 | -0.15 |  |
| **Interpersonal Constraints** |  |  |  |  |
| The people I know don’t have time to fish | 0.91* | -0.08 | -0.91 |  |
| The people I know don’t have money to fish | 0.90* | 0.36 | -0.78 |  |
| The people I know are not interested in fishing | 0.91* | 0.17 | -0.86 |  |
| The people I know don’t feel it’s appropriate to fish | 0.89* | 0.49 | -0.62 |  |
| I don’t know other people I can fish with | 0.89* | 0.43 | -0.82 |  |
| **Intrapersonal Constraints** |  |  |  |  |
| I have a lack of information about fishing opportunities in Illinois | 0.90* | 0.37 | -0.77 |  |
| I have difficulty understanding fishing regulations | 0.89* | 0.50 | -0.66 |  |
| I have a lack of interest in fishing | 0.80* | 0.96 | -0.11 |  |
| **Structural Constraints** |  |  |  |  |
| Fishing facilities are too crowded | 0.91* | 0.11 | -0.76 |  |
| I have too many family and/or work commitments | 0.91* | -0.17 | -1.02 |  |
| There are not enough fish for me to catch | 0.89* | 0.43 | -0.70 |  |
| I don’t have access to fishing opportunities | 0.90* | 0.33 | -0.88 |  |
| The price of a fishing license is too high | 0.91* | 0.12 | -0.88 |  |
| There is not enough shoreline access | 0.91* | 0.15 | -0.92 |  |
| There are not enough boat ramps | 0.91* | 0.20 | -0.77 |  |
| Parking is too expensive or not available | 0.91* | 0.23 | -0.88 |  |
| Other activities take up my time that could be spent fishing | 0.91* | -0.26 | -0.91 |  |
| **Environmental Identity** |  |  |  |  |
| I like to spend time outdoors in natural settings (such as woods, mountains, rivers, parks, lakes or beaches, gardens) | 0.79* | -0.97 | 1.04 |  |
| I think of myself as a part of nature, not separate from it | 0.84* | -0.71 | -0.13 |  |
| I feel comfortable out in nature | 0.79* | -1.05 | 0.71 |  |
| When I am upset or stressed, I can feel better by spending some time outdoors surrounded by nature | 0.79* | -1.09 | 0.86 |  |
| I enjoy encountering elements of nature, like trees and grass, even when I am in a city setting | 0.79* | -1.08 | 1.00 |  |
| Learning about the natural world should be part of everyone’s upbringing | 0.80* | -0.98 | 0.41 |  |
| If I could choose, I would prefer to live where I can have a view of the natural environment, such as trees or fields | 0.81* | -0.99 | 0.57 |  |
| An important part of my life would be missing if I was not able to get outside and enjoy nature from time to time | 0.80* | -0.98 | 0.44 |  |
| I feel refreshed when I spend time in nature | 0.77* | -1.11 | 1.22 |  |

^a^**p* < .05; indicates a non-normal distribution

^b^Results were calculated using IBM Statistical Package for the Social Sciences
