# Supplementary material for: Fishing Participation Explained Through an Extended Theory of Planned Behavior Model
Source: Environ Manage. 2026 Apr 30;76(6):179. doi: 10.1007/s00267-026-02482-5 (PMC13133205; doi:10.1007/s00267-026-02482-5)
Supplement: Supplementary file 2 — R3_questionnaire [file 267_2026_2482_MOESM2_ESM.docx]

**This is a survey about fishing participation in Illinois**

The University of Illinois at Urbana-Champaign is conducting research to learn more about the perspectives of recreational anglers in Illinois. We are asking you to participate in this study so we can provide decision-makers with insights on the reasons why you go fishing, your beliefs, and your behaviors related to the environment. This survey will take approximately 15 minutes. Your participation is voluntary. If you decide to participate, you are free to withdraw at any time. If for any reason you prefer not to participate in this study, you may exit now.

Those of us at the University of Illinois who may see your information will maintain confidentiality to the extent of laws and university policies. Personal identifiers will not be published or presented. If you have questions or concerns about your rights as a participant, please contact the University of Illinois at Urbana-Champaign Office for the Protection of Research Subjects at 217-333-2670 or via email at [irb@illinois.edu](mailto:irb@illinois.edu). If you have any questions about the study, please contact the project leader, Carena van Riper at [cvanripe@illinois.edu](mailto:cvanripe@illinois.edu).

To participate in this study, you must be 18 years of age or older, reside in Illinois, and have gone fishing in Illinois at least once in the past 5 years.

We appreciate your careful consideration of each question. Please offer any comments you might have at the end of the survey.

Please check the following boxes to indicate your qualifications to participate in the study.

- I have read and understand the above information
- I certify that I am 18 years or older
- I certify that I am physically present in the United States
- I have gone fishing in Illinois at least once in the past five years

We care about the quality of our survey data and hope to receive the most accurate measures of your opinions, so it is important to us that you thoughtfully provide your best answer to each question in the survey. Do you commit to providing your thoughtful and honest answers to the questions in this survey?

| - - - I will provide my best answers |
| --- |
| - - - I will not provide my best answers |
| - - - I cannot promise either way |

By checking the box, I indicate my willingness to voluntarily take part in this survey.


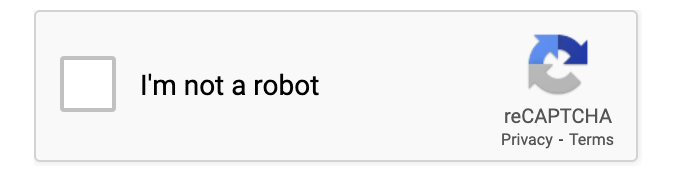


# Section 1 of 4: Background Information

*In this section, we ask you to provide information about your fishing experiences.*

1. About how many days have you gone fishing in the past 12 months? __________
2. About how many years of fishing experience do you have? __________
3. For each of the following years, please indicate how often you went fishing. [Dropdown menu provided for 2019-2023 with the following response scale]

|  |  |  |  |  |
| --- | --- | --- | --- | --- |
| Never | A few times | About once a month | About once a week | More than once a week |

1. Please rate your intention to make time for fishing in the next 12 months.

|  |  |  |  |  |
| --- | --- | --- | --- | --- |
| Not at all intend | Somewhat intend | Moderately intend | Strongly intend | Very strongly intend |

1. How would you rate your skills in relation to other anglers?

|  |  |  |  |  |
| --- | --- | --- | --- | --- |
| Much lower than average | Lower than average | Average | Higher than average | Much higher than average |

1. Have you purchased a fishing license in Illinois between 2019 and 2023?

| - - - No |
| --- |
| - - - Yes ⮊ If yes, respondent is prompted to enter the total number of years |

1. Where have you spent most of your time fishing in Illinois?

| - - - Inland lakes and ponds |
| --- |
| - - - Rivers and streams |
| - - - Lake Michigan |
| - - - I spend about an equal amount of time fishing from the above options |

1. What is your most commonly used fishing mode?

| - - - Fishing from the shoreline or dock (including wading) |  |
| --- | --- |
| - - - Fishing from a boat |  |
| - - - I spend about an equal amount of time fishing from boats and shorelines |  |
| 8a. If yes to 8b or 8c, what kind of boat do you use most often? | |
| - - - Kayak or canoe |  |
| - - - Pontoon boat |  |
| - - - Motorboat |  |

1. Which species do you fish for most often? (*✓* all that apply)

| - - - Atlantic salmon | - - - Bluegill | - - - Brook trout |
| --- | --- | --- |
| - - - Brown trout | - - - Carp | - - - Catfish |
| - - - Chinook / king salmon | - - - Coho salmon | - - - Crappie |
| - - - Drum / sheepshead | - - - Gar | - - - Lake trout |
| - - - Largemouth bass | - - - Muskie | - - - Northern pike |
| - - - Rainbow trout / steelhead | - - - Smallmouth bass | - - - Walleye |
| - - - White bass | - - - Whitefish | - - - Yellow perch |
| - - - Other (please list): **_______________** | | |

| 9b. We would like to know how important catching fish to eat is to you. Please rate how strongly you agree with the following three statements. | Strongly disagree | Somewhat disagree | Neither agree nor disagree | Somewhat agree | Strongly agree |
| --- | --- | --- | --- | --- | --- |
| I usually eat the fish that I catch. |  |  |  |  |  |
| I happier if I release the fish that I catch. |  |  |  |  |  |
| I never eat the fish that I catch. |  |  |  |  |  |

1. Where have you learned about issues related to fishing in Illinois? (*✓* all that apply)

| - - - Retailers | - - - Government officials | - - - Friends and family |
| --- | --- | --- |
| - - - Scientists | - - - Fishing organizations | - - - Fishing guides |
| - - - News organizations |  |  |

| 11. We would like your input on the best ways of disseminating information. How useful would the following resources be for you to learn about fishing in Illinois? | Not at all useful | Slightly useful | Moderately useful | Very useful | Extremely useful |
| --- | --- | --- | --- | --- | --- |
| a. Educational signs at fishing sites |  |  |  |  |  |
| b. Booths at fishing related events |  |  |  |  |  |
| c. Newspaper, magazine, or newsletter articles |  |  |  |  |  |
| d. Television programs |  |  |  |  |  |
| e. Radio programs |  |  |  |  |  |
| f. Podcasts |  |  |  |  |  |
| g. Illinois Department of Natural Resources website |  |  |  |  |  |
| h. Brochures and handouts |  |  |  |  |  |
| i. Webinars |  |  |  |  |  |
| j. Scholarly articles |  |  |  |  |  |
| k. Public meetings about fishing |  |  |  |  |  |
| l. Illinois fishing information guide (fishing digest) |  |  |  |  |  |
| m. Outdoor Illinois Journal |  |  |  |  |  |
| n. Facebook |  |  |  |  |  |
| o. Twitter or “X” |  |  |  |  |  |
| p. YouTube videos |  |  |  |  |  |
| q. TikTok |  |  |  |  |  |

# Section 2 of 4: The Meaning of Fishing

*In this section, we ask you to think of the importance of fishing among your family, friends, and social groups. This information will help decision makers understand what fishing means to you.*

| 12. These questions are about the role fishing played in your life. How strongly you agree or disagree with the following statements? | Strongly Disagree | Disagree | Neutral | Agree | Strongly Agree |
| --- | --- | --- | --- | --- | --- |
| a. Growing up, fishing was treated as an important activity in  my family |  |  |  |  |  |
| b. In my childhood home, time was set aside to go fishing |  |  |  |  |  |
| c. Fishing was an opportunity to spend quality time with family  members when I was younger |  |  |  |  |  |
| d. My family believed that fishing taught important skills |  |  |  |  |  |
| e. Fishing is considered a respectable pastime within society |  |  |  |  |  |
| f. I find that society is generally accepting of fishing as a  practice |  |  |  |  |  |
| g. Fishing is seen as an admirable skillset in society |  |  |  |  |  |
| h. Many people in society would enjoy talking about  fishing |  |  |  |  |  |

| 13. These questions are about the relationship you and the people around you have with fishing. How strongly do you agree or disagree with the following statements? | Strongly Disagree | Disagree | Neutral | Agree | Strongly Agree |
| --- | --- | --- | --- | --- | --- |
| a. The people that I know through fishing are important to me |  |  |  |  |  |
| b. I would spend less time with some of the people I know if I  were to stop fishing |  |  |  |  |  |
| c. Fishing is a way to spend time with friends and family |  |  |  |  |  |
| d. I would miss out on important social experiences if I were to  stop fishing |  |  |  |  |  |
| e. Fishing plays a major role in my life |  |  |  |  |  |
| f. Fishing gives me a sense of fulfillment |  |  |  |  |  |
| g. Fishing is very important to me |  |  |  |  |  |
| h. I find fishing satisfying |  |  |  |  |  |
| i. Fishing is a part of my personal identity |  |  |  |  |  |
| j. I would feel a sense of loss if I were not able to fish |  |  |  |  |  |
| k. Fishing is part of who I am |  |  |  |  |  |

14. How important do you consider making time for fishing trips in the next 12 months?

|  |  |  |  |  |
| --- | --- | --- | --- | --- |
| Not at all important | Slightly important | Moderately important | Very important | Extremely important |

# Section 3 of 4: Beliefs and Barriers to Fishing

| 15. We would like to understand the personal and social reasons why you go fishing. How strongly do you agree or disagree with the following statements? | Strongly Disagree | | Disagree | | Neutral | Agree | | Strongly Agree |
| --- | --- | --- | --- | --- | --- | --- | --- | --- |
| a. Fishing offers many benefits to me | |  | |  |  | |  |  |
| b. Fishing is generally a pleasant experience | |  | |  |  | |  |  |
| c. The practice of fishing teaches me useful skills | |  | |  |  | |  |  |
| e. If I engage in fishing, people who are important to me would  approve | |  | |  |  | |  |  |
| f. Most people who are important to me think that participation  in fishing is desirable | |  | |  |  | |  |  |
| g. The people that I know are encouraging of my participation  in fishing | |  | |  |  | |  |  |

*In this section we ask about your beliefs about fishing and the factors that might prevent you from fishing in the future.*

Ass

This is an attention check question. Please select “strongly agree”.

|  |  |  |  |  |
| --- | --- | --- | --- | --- |
| Strongly disagree | Somewhat disagree | Neither agree nor disagree | Somewhat agree | Strongly agree |

| 16. We would like to know how easy it is for you to go fishing. How strongly do you agree or disagree with the following statements? | Strongly Disagree | Disagree | Neutral | Agree | Strongly Agree |
| --- | --- | --- | --- | --- | --- |
| a. Whether or not I can go fishing is largely within my own control |  |  |  |  |  |
| b. For me, going fishing is easy |  |  |  |  |  |
| c. I can easily find time to go fishing |  |  |  |  |  |
| d. I believe I have the ability to go fishing as much as I want |  |  |  |  |  |

| 17. We would like to understand some of the reasons why you might not be able to go fishing. How strongly do you agree or disagree with the following statements? | Strongly Disagree | Disagree | Neutral | Agree | Strongly Agree |
| --- | --- | --- | --- | --- | --- |
| a. Fishing facilities are too crowded |  |  |  |  |  |
| b. I have too many family and/or work commitments |  |  |  |  |  |
| c. There are not enough fish for me to catch |  |  |  |  |  |
| d. I don’t have access to fishing opportunities |  |  |  |  |  |
| e. The price of fishing licenses is too high |  |  |  |  |  |
| f. There is not enough shoreline access |  |  |  |  |  |
| g. There are not enough boat ramps |  |  |  |  |  |
| h. Parking is too expensive or not available |  |  |  |  |  |
| i. Other activities take up my time that could be spent fishing |  |  |  |  |  |
| j. The people I know don’t have time to fish |  |  |  |  |  |
| k. The people I know don’t have money to fish |  |  |  |  |  |
| l. The people I know are not interested in fishing |  |  |  |  |  |
| m. The people I know don’t feel it’s appropriate to fish |  |  |  |  |  |
| n. I don’t know other people that I can fish with |  |  |  |  |  |
| o. I have a lack of information about fishing opportunities in  Illinois |  |  |  |  |  |
| p. I have difficulty understanding Illinois fishing regulations |  |  |  |  |  |
| q. I have limited fishing skills |  |  |  |  |  |
| r. I have a lack of interest in fishing |  |  |  |  |  |

18. Are there any additional barriers not listed in the previous question that have prevented you from going fishing? If so, please list them here. ________________________________________________________________________________________________________________________

19. How strongly do you intend to make time for fishing over the next 12 months?

|  |  | |  |  |  |  |
| --- | --- | --- | --- | --- | --- | --- |
| Not at all | | | Somewhat | Moderately | Very | Extremely |

| 20. These questions are about the reasons why you fish. Please rate the extent to which each of the following statements describes your motivation to go fishing. | Not at all true of me | Slightly true of me | Neither true nor untrue of me | Moderately true of me | Completely true of me |
| --- | --- | --- | --- | --- | --- |
| a. To be where things are natural |  |  |  |  |  |
| b. To view the scenic beauty |  |  |  |  |  |
| c. To be close to nature |  |  |  |  |  |
| d. To enjoy the smells and sounds of nature |  |  |  |  |  |
| e. To be where it is quiet |  |  |  |  |  |
| f. To experience solitude |  |  |  |  |  |
| g. To experience the peace and calm |  |  |  |  |  |
| h. To experience new and different things |  |  |  |  |  |
| i. To discover something new |  |  |  |  |  |
| j. To explore the area |  |  |  |  |  |
| k. To do something with my family |  |  |  |  |  |
| l. To bring my family closer together |  |  |  |  |  |
| m. To be with friends |  |  |  |  |  |
| n. To be with people who enjoy the same things I do |  |  |  |  |  |
| o. To learn what I am capable of |  |  |  |  |  |
| p. To develop my skills and abilities |  |  |  |  |  |
| q. To gain a sense of self-confidence |  |  |  |  |  |
| r. For the challenge or sport of fishing |  |  |  |  |  |
| s. For monetary gain (such as tournament prizes) |  |  |  |  |  |
| t. To think about good times that I have had in the past |  |  |  |  |  |
| u. For the sake of tradition |  |  |  |  |  |
| v. For food |  |  |  |  |  |

| 21. These questions are about your relationship with the environment. Please rate the extent to which each of the following statements describes you. | Not at all true of me | Slightly true of me | Neither true nor untrue of me | Moderately true of me | Completely true of me |
| --- | --- | --- | --- | --- | --- |
| a. I like to spend time outdoors in natural settings (such as woods, mountains, rivers, parks, lakes, beaches, gardens) |  |  |  |  |  |
| b. I think of myself as a part of nature, not separate from it |  |  |  |  |  |
| c. If I had enough resources such as time or money, I would  spend some of them to protect the natural environment |  |  |  |  |  |
| d. When I am upset or stressed, I can feel better by spending  some time outdoors surrounded by nature |  |  |  |  |  |
| f. Behaving responsibly toward nature – living a sustainable  lifestyle – is important to who I am |  |  |  |  |  |
| g. Learning about the natural world should be part of everyone’s  upbringing |  |  |  |  |  |
| h. If I could choose, I would prefer to live where I can have a  view of the natural environment, such as trees or fields |  |  |  |  |  |
| i. An important part of my life would be missing if I was not able  to get outside and enjoy nature from time to time |  |  |  |  |  |
| k. I feel refreshed when I spend time in nature |  |  |  |  |  |

# Section 4 of 4: About You

| 22. What is your gender? | - - - Male | - - - Female | - - - Other |
| --- | --- | --- | --- |

23. In what year were you born? ____________________

24. What is your annual household income (in USD) before taxes? (Please *✓* one)

| - - - Less than $24,999 | - - - $25,000-$49,999 | | - - - $50,000-$74,999 |
| --- | --- | --- | --- |
| - - - $75,000-$99,999 | - - - $100,000-$124,999 | | - - - $125,000-$149,999 |
| - - - $150,000-$174,999 | - - - $175,000-$199,999 | | - - - $200,000 and over |
| - - - Prefer not to answer | |  |  |

25. What is the highest level of education you have completed? (Please *✓* one)

| - - - Less than high school | - - - Some high school | - - - High school graduate or GED |
| --- | --- | --- |
| - - - Associate’s degree | - - - Bachelor’s degree | Graduate degree (MA, MS, PhD, JD, MD, etc.) |

26. With which racial or ethnic group(s) do you identify? (Please *✓* all that apply)

| - - - White | - - - Black or African American | - - - Hispanic or Latino |
| --- | --- | --- |
| - - - Asian | - - - Native Hawaiian or Pacific Islander | - - - Other (please list): ______________ |

27. What is your zip code? ________________________

# Thanks for your participation!

**If you have any additional thoughts about this study that were not reflected in the questions above, please share them here.**

Carena van Riper, Ph.D.

Department of Natural Resources and Environmental Sciences

University of Illinois at Urbana-Champaign

Phone: 217-244-9317

Email: cvanripe@illinois.edu
